# Relationship between neural network structure and temperament/personality traits in healthy subjects

**DOI:** 10.1192/j.eurpsy.2023.1164

**Published:** 2023-07-19

**Authors:** Y. Kawasaki, Y. Koide, A. Ohata, T. Senoh, Y. Kataoka, T. Shimada, T. Nagasawa, T. Uehara

**Affiliations:** Neuropsychiatry, Kanazawa Medical UNiversity, Uchinada, Ishikawa, Japan

## Abstract

**Introduction:**

Cloninger divides personality into temperament and character, proposing that temperament is innate and character is shaped by environment. With the development of noninvasive methods for measuring central nervous system activity, there have been many attempts to test personality theories using neuroscientific research methods. Thus, the use of neuroscience to examine existing theories of personality will enable a review of these theories and may lead to the formulation of new theories of personality.

**Objectives:**

The purpose of this study was to investigate the biological factors underlying temperament and personality development in healthy adults by analyzing neural networks in the brain using resting-state functional magnetic resonance imaging.

**Methods:**

The study was conducted after obtaining prior approval from the Ethics Committee of Kanazawa Medical University. Eighty-one healthy subjects who consented to the study after explaining the purpose and methods were imaged with a 3T MRI scanner in the resting state, and statistical image analysis was performed using the CONN toolbox. Personality and temperament were assessed using the temperament personality test based on Cloninger’s 7-dimensional model of personality.

**Results:**

Five types of neural networks were extracted by independent component analysis, including Salience, Default mode, and Language. Regression analysis revealed a significant relationship between the functional connectivity of the networks and temperament/personality traits.

**Conclusions:**

We were able to observe the functional connectivity of representative neural networks from the data of healthy subjects, suggesting that individual differences in the degree of functional connectivity of neural networks may be related to the individual characteristics of temperament and personality of the subjects.

**Disclosure of Interest:**

None Declared

